# Risk factors for anxiety and its impacts on acute exacerbation in older patients with chronic obstructive pulmonary disease

**DOI:** 10.3389/fmed.2024.1340182

**Published:** 2024-04-05

**Authors:** Yan Mou, Lin Shan, Yunhuan Liu, Yue Wang, Zhengming He, Xiangyang Li, Huili Zhu, Haiyan Ge

**Affiliations:** Department of Pulmonary and Critical Care Medicine, Huadong Hospital, Fudan University, Shanghai, China

**Keywords:** COPD, anxiety, Hamilton Anxiety Rating Scale (HAMA), comorbidity, acute exacerbation

## Abstract

**Background:**

Anxiety is common in patients with chronic obstructive pulmonary disease (COPD), especially in older patients with the definition of age over 60 years old. Few studies have focused on anxiety in older COPD patients. This study aimed to analyze the risk factors of anxiety in older COPD patients and the impacts of anxiety on future acute exacerbation.

**Methods:**

The general information, questionnaire data, previous acute exacerbation and pulmonary function were collected. Hamilton Anxiety Rating Scale (HAMA) was used to evaluate the anxiety of older COPD patients. The patients were followed up for one year, the number and the degrees of acute exacerbations of COPD were recorded.

**Results:**

A total of 424 older COPD patients were included in the analysis. 19.81% (*N* = 84) had anxiety symptoms, and 80.19% (*N* = 340) had no anxiety symptoms. There were increased pack-years, more comorbidities, and more previous acute exacerbations in older COPD patients with anxiety compared to those without anxiety (*P* < 0.05). Meanwhile, a higher modified Medical Research Council (mMRC), a higher COPD assessment test (CAT) score and a shorter six-minute walking distance (6MWD) were found in older COPD patients with anxiety (*P* < 0.05). The BODE index, mMRC, CAT score, comorbidities and acute exacerbations were associated with anxiety. Eventually, anxiety will increase the risk of future acute exacerbation in older COPD patients (OR = 4.250, 95% CI: 2.369–7.626).

**Conclusion:**

Older COPD patients with anxiety had worsening symptoms, more comorbidities and frequent acute exacerbation. Meanwhile, anxiety may increase the risk of acute exacerbation in the future. Therefore, interventions should be provided to reduce the risk of anxiety in older COPD patients at an early stage.

## 1 Introduction

Chronic obstructive pulmonary disease (COPD) is one of the most frequent respiratory diseases among middle-aged and old individuals, contributing to significant global morbidity and mortality (1). The annual death toll associated with COPD reaches approximately 3 million, and it is predicted to rise to over 4.5 million by 2030 worldwide (2). There are currently 99.9 million people with COPD in China, and the prevalence of COPD in people over 40 years old and over 60 years old are 13.7 and 27%, respectively (3). With the increasing levels of air pollution and aging population, COPD is expected to become the primary economic burden of chronic diseases in the future (4). Therefore, it is crucial for the society to display special concern on COPD.

Recently, there has been growing attention toward comorbidities in individuals with COPD (5). Comorbidity prevalence is quite high among COPD patients: more than half have one or two comorbidities; while around 15.8% have three or four comorbidities; additionally, about 6.8% suffer from five or more comorbidities (6). Anxiety is a common comorbidity observed in individuals with COPD. In the general adult population of China, anxiety was found to have a prevalence of 5.3% according to the Hospital Anxiety and Depression Scale (HADS) and 5.6% based on the Diagnostic and Statistical Manual of Mental Disorders fourth edition (DSM-IV) scale (7, 8). The prevalence of anxiety ranged from 10 to 55% for inpatients and 13–46% for outpatients among patients with COPD (9).

Patients with COPD often experience poor mental health and older COPD patients are more likely to develop mental health especially anxiety (10). There are many risk factors for anxiety in COPD patients, such as continued smoking, poor knowledge, loneliness, and low social status (11, 12). As COPD progressing and age increasing, patients experience increased dyspnea, decreased physical function, and limited physical and social activities which leads to more severe anxiety symptoms (13). They often faced accelerated health deterioration, increased risk of adverse events, reduced quality of life, and experienced frequent acute exacerbation (14). However, there have been limited clinical trials conducted in this age group. In this study, we attempted to identify the risk factors of anxiety in older COPD patients and the impacts of anxiety on future acute exacerbation.

## 2 Materials and methods

### 2.1 Study design and participants

This study involved 424 older COPD patients who visited pulmonary outpatient clinic at fifteen hospitals in Shanghai from June 2017 to December 2020 (ChiCTR2000030911). All the patients were in a stable condition and randomly admitted. Written informed consent was obtained. The study was approved by the Ethics Committee of Huadong Hospital.

Inclusion criteria were as follows: (1) primary diagnosis of COPD according to the Global Initiative for Chronic Obstructive Lung Disease (GOLD) criteria (1). The forced expiratory volume in the first second of forced vital capacity (FEV1/FVC) < 0.7 after inhaling bronchodilators (BD) confirmed persistent airflow limitation (15); (2) participants with age ≥ 60 years; (3) signed written informed consent in the study. Exclusion criteria were as follows: (1) history of COPD acute exacerbation within one month prior to enrollment; (2) history of respiratory infection within one month prior to enrollment; (3) mental disorders (e g, schizophrenia, cognitive disorder, senile dementia, or Alzheimer) impairing capacity for informed consent; (4) missing follow-up information.

### 2.2 Demographic data

All COPD patients were required to complete a structured questionnaire and were given a thorough physical examination. All data were collected by physicians. The frequency and severity of acute exacerbation in the previous year were recorded at the first visit. Patients were followed up for one year. Demographic characteristics and clinical features were recorded. Comorbidity included diseases of respiratory system (asthma, allergic rhinitis, lung cancer, pulmonary embolism and bronchiectasis), cardiovascular system (angina, arrhythmia, hypertension and heart failure), metabolism system (diabetes, osteoporosis and metabolism syndrome), nervous system (stroke, subarachnoid and dementia), digestive system (peptic ulcer, digestive tumor and liver disease) and other diseases like connective tissue disease, peripheral vascular disease, lymphoma, leukemia, and anxiety.

### 2.3 Assessment of anxiety

The Hamilton Anxiety Rating Scale (HAMA) was widely used to screen anxiety in the general hospital. All participants were assessed by the same physician. All items of the HAMA were scored on a scale of 0–4 points. The HAM-A included 14 items covering two types of symptom factors which were psychic anxiety factors and somatic anxiety factors. The psychic anxiety factors were as follows: anxiety mood, tension, fears, insomnia, difficulties in concentration and memory, depression mood and behaviors during the interview. The somatic anxiety factors included somatic symptoms concerning seven symptoms: muscle, sensory, cardiovascular, respiratory, gastrointestinal, genito-urinary and other autonomic nervous system symptoms. HAMA ≥ 14 was defined as COPD with anxiety (16).

### 2.4 Definition of acute exacerbation

An acute exacerbation of chronic obstructive pulmonary disease (AECOPD) defines as an acute worsening of respiratory symptoms that result in additional therapy (1). Exacerbation events are classified as mild [treated with short acting bronchodilators (SABDs) only], moderate (relieved by SABDs plus antibiotics, with or without oral corticosteroids), or severe (refer to acute exacerbation requiring hospitalization, emergency admission or ICU transferring) (17). The number of total exacerbations, mild, moderate, or severe exacerbations in the previous year and in the following-up one year were documented.

### 2.5 Assessment of pulmonary function

Spirometry was obtained from a Jaeger Toennies spirometer (Höchberg, Germany) according to the American Thoracic Society (ATS) guidelines (1). Each patient completed the spirometry test and bronchodilator reversibility test (BDR). The parameters including FEV1/predicted post BD, FEV1/FVC post BD and residual volume/total lung capacity (RV/TLC) % were recorded. The spirometry tests were performed by professional technicians and the results were interpreted by two physicians. COPD severity was evaluated according to the severity of airflow obstruction. GOLD1: FEV1 ≥ 80% predicted means mild; GOLD2: 50% ≤ FEV1 < 80% predicted means moderate; GOLD3: 30% ≤ FEV1 < 50% predicted means severe; GOLD4: FEV1 ≤ 30% predicted means very severe.

### 2.6 Assessment of COPD symptoms and health-related quality of life

The BODE index, a multidimensional grading system, is based on the body-mass index (B), the degree of airflow obstruction (O) evaluated by FEV1, the grade of dyspnea (D) assessed by the modified Medical Research Council (mMRC) dyspnea score, and the exercise capacity (E) assessed by the six-minute walking distance test (6MWD). The total scores of the BODE index ranged from 0 to 10 points (higher scores indicated more severity). The BODE index predicted death and other poor outcomes in COPD (18). mMRC dyspnea score was used to estimate the impact of dyspnea in everyday activities. The COPD assessment test (CAT) and St. George’s respiratory questionnaire (SGRQ) were used to evaluate health-related quality of life (HRQL) (19). 6MWD was carried out to evaluate exercise capacity of COPD patients (20). The evaluation was done by professional physicians.

### 2.7 Statistical analyses

All statistical analyses were performed by a commercially software program (SPSS 22.0 for Windows; SPSS, Chicago, IL, USA). Continuous variates were presented as mean ± standard deviation for the normally distributed data or median (25th and 75th percentile) for the non-normally distributed data, while categorical variates were presented as *n* or *n* (%). Student’s *t*-test was used for normally distributed data, while the Mann Whitney u test was used for non-normally distributed data. The categorical variates were analyzed by chi-square test. We used Logistic regression to evaluate risk factors of anxiety in COPD patients. We used Logistic regression and Poisson regression to predict the effect of anxiety on future exacerbation. *P* < 0.05 was considered statistically significant.

## 3 Results

### 3.1 General characteristics of older COPD patients

A total of 424 older COPD patients were included to analyze the relation between anxiety and its associated factors in older COPD patients. There were 380 (89.60%) males and 44 females (10.40%) with a median age of 70 years. The median pack-years were 30 (15–50). The median FEV1/predict post BD was 31.30 (28.00–52.40) %. The median score on SGRQ was 36 (31.00–51.75), while the median 6MWD (m) was 310 (295.50–340.00). Among the 424 patients, 86.79% had one or more comorbidities, and 56.13% had at least one exacerbation in the previous one year (Table 1).

**TABLE 1:** Baseline characteristics of the 424 subjects.

Characteristics	COPD
Age (years) [M (P25, P75)]	70 (64–78)
Gender (male) [*n* (%)]	380 (89.60)
BMI (kg/m2) [M (P25, P75)]	23.52 (22.03–24.91)
**Pack-years [M (P25, P75)]**	
Never smoker	NA
Former/current smoker	30 (15–50)
COPD course (years) [M (P25, P75)]	8 (5–10)
**mMRC**	
Grade 0 [*n* (%)]	29 (6.8)
Grade 1 [*n* (%)]	156 (36.8)
Grade 2 [*n* (%)]	139 (32.8)
Grade 3 [*n* (%)]	82 (19.3)
Grade 4 [*n* (%)]	18 (4.2)
SGRQ [M (P25, P75)]	36 (31.00–51.75)
CAT score [M (P25, P75)]	11.5 (8.25–17.00)
6 MWD (m) [M (P25, P75)]	310 (295.50–340.00)
**Pulmonary function**	
FEV1/predict post BD (%)[M (P25, P75)]	31.30 (28.00–52.40)
FEV1/FVC post BD (%)[M (P25, P75)]	63.00 (51.00–66.50)
RV/TLC% [M (P25, P75)]	43.00 (41.00–108.00)
Comorbidities [*n* (%)]	368 (86.79)
Previous exacerbations [*n* (%)]	238 (56.13)

COPD, chronic obstructive pulmonary disease; BMI, body mass index; FEV1, forced expiratory volume in 1 s; BD, bronchodilator; FVC, forced vital capacity; CAT, the COPD assessment test; mMRC, modified Medical Research Council; SGRQ, St. George’s respiratory questionnaire; 6MWD, six-minute walking distance; NA, not applicable.

### 3.2 Comparison of baseline data between older COPD patients with anxiety and older COPD patients without anxiety

Older COPD patients in our analysis were divided into two groups (Table 2). Group 1, Older COPD patients with anxiety; Group 2, Older COPD patients without anxiety. Table 2 showed the baseline data and comparisons between the two groups. Group 1 included 84 older COPD patients with anxiety: 76 (90.48%) males and 8 females (9.52%) were included, while the average age was 68 (63–77) years. Group 2 consisted of 340 older COPD patients without anxiety: 304 (89.41%) males and 36 females (10.59%) were included, while the average age was 70 (65–78) years. There were increased pack-years, more comorbidities, and more acute exacerbations in older COPD patients with anxiety. They were statistically different. There were statistically differences in mMRC, CAT score and 6MWD between the two groups. However, there were no statistically differences in gender, BMI, COPD courses, SGRQ score between the two groups.

**TABLE 2:** Comparison of baseline data between COPD with anxiety and COPD without anxiety.

Characteristics	Group 1 COPD with anxiety	Group 2 COPD without anxiety	*p*-value
Number of subjects [*n* (%)]	84 (19.81)	340 (80.19)	–
Gender (male/female) [*n*]	76/8	304/36	0.775
Age (years) [M (P25, P75)]	68 (63–77)	70 (65–78)	0.263
BMI (kg/m2) [M (P25, P75)]	23.41 (21.54–24.42)	23.61 (22.06–25.00)	0.314
Pack-years [M (P25, P75)]	40 (22.50–57.25)	30 (12.50–47.13)	0.008*
COPD courses (years) [M (P25, P75)]	8 (3–12)	8 (6–10)	0.517
**mMRC**			
Grade 0 [*n* (%)]	0 (0)	29 (8.53)	< 0.0001*
Grade 1 [*n* (%)]	13 (14.94)	143 (42.06)	
Grade 2 [*n* (%)]	35 (41.67)	104 (30.59)	
Grade 3 [*n* (%)]	24 (28.57)	58 (17.06)	
Grade 4 [*n* (%)]	12 (14.29)	6 (1.76)	
SGRQ score [M (P25, P75)]	37.5 (27.50–45.75)	36 (31.00–65.00)	0.073
CAT score [M (P25, P75)]	17.5 (13.75–22.25)	11 (8–14)	< 0.0001*
6 MWD (m) [M (P25, P75)]	276 (210–336)	320 (300–362)	< 0.0001*
**Comorbidity**			
One or no comorbidity [*n* (%)]	19 (22.62)	204 (60.00)	< 0.0001*
More than one comorbidity [*n* (%)]	65 (77.38)	136 (40.00)	
**Exacerbation**			
With exacerbation [*n* (%)]	68 (80.95)	170 (50.00)	< 0.0001*
Without exacerbation [*n* (%)]	16 (19.05)	170 (50.00)	

COPD, chronic obstructive pulmonary disease; BMI, body mass index; SGRQ, St. George’s respiratory questionnaire; mMRC, modified Medical Research Council. CAT, the COPD assessment test; 6MWD, six-minute walking distance.

*Results were considered significant when *p* < 0.05.

### 3.3 Possible factors of anxiety in terms of symptoms, disease severity, and exercise capacity

Higher COPD severity evaluated by BODE index was associated with a higher risk of anxiety in older COPD patients. Degree of dyspnea, evaluated by mMRC had association with the risk of anxiety. The higher the CAT score, the higher the risk of anxiety. CAT score in severe and very severe COPD was 3.547 times of that in mild and moderate COPD. However, 6MWD were not related to anxiety (Table 3).

**TABLE 3:** Possible factors associated with anxiety in older COPD patients.

	Risk of anxiety	
**Characteristics**	**Unadjusted OR (95% CI)**	**Multivariable adjusted OR (95% CI)**
BODE index	2.492 (1.828–3.397)*	2.493 (1.828–3.398)*
mMRC	2.417 (1.839–3.176)*	2.462 (1.860–3.259)*
CAT score	3.63 (2.212–5.959)*	3.547 (2.129–5.909)*
6MWD (m)	0.991 (0.987–0.995)	0.990 (0.986–0.995)

Results are from logistic regression analysis of relations between possible factors associated with anxiety. Multivariable adjustment included age, gender, BMI, COPD course and smoking history.

*Results were considered significant when *p* < 0.05.

### 3.4 Related factors of anxiety in terms of comorbidities and acute exacerbations

Older COPD patients with more than one comorbidity had greater risk of anxiety than those with none or one comorbidity with the odds ratio of 5.671 (95% CI: 3.193–10.07). Compared with older COPD patients without acute exacerbation in the previous year, the odds ratio of anxiety in older COPD patients with acute exacerbation was 4.004 (95% CI: 2.204–2.273) (Table 4).

**TABLE 4:** Relations between comorbidities and exacerbations and anxiety in older COPD patients.

Indicators	Unadjusted OR (95% CI)	*p*-value	Multivariable adjusted OR (95% CI)	*p*-value
Comorbidities ≤ 1	1.00	–	1.00	–
> 1	5.132 (2.945–8.942)	*p* < 0.001*	5.671 (3.193–10.07)	*p* < 0.001*
Exacerbations < 1	1.00	–	1.00	–
≥ 1	4.25 (2.369–7.626)	*p* < 0.001*	4.004 (2.204–7.273)	*p* < 0.001*

Results are from logistic regression analysis of relations between comorbidities, previous exacerbations and the risk of anxiety. Results were expressed as odds ratio (95% confidence interval). Multivariable adjustment included age, gender, BMI, COPD course and smoking history.

*Results were considered significant when *p* < 0.05.

### 3.5 Anxiety associated with the increased risk of future acute exacerbation

Anxiety was associated with increased risk of various degrees of future exacerbation from the aspect of both incidence and frequency. Unadjusted odds ratio (95% CI) of future exacerbation for older COPD patients with anxiety was 4.250 (2.369–7.626) compared to those without anxiety. Corresponding unadjusted odds ratios (95% CI) were 2.653 (1.526–4.611) and 2.006 (1.221–3.297) for moderate and severe exacerbations in one year (Table 5). Meanwhile, Unadjusted incidence-rate ratio (95% CI) for total acute exacerbation was 2.000 (1.572–2.545) in older COPD patients with anxiety compared to those without anxiety. Corresponding incidence-rate ratios (95% CI) were 2.285 (1.422–3.669) and 2.080 (1.350–3.203) for moderate and severe acute exacerbations within one year (Table 6).

**TABLE 5:** Anxiety in relation to incidence of different levels of acute exacerbations in COPD.

	Incidence of exacerbation in 12 months							
**Anxiety**			**Unadjusted OR (95% CI)**				**Multivariable adjusted OR (95% CI)**	
	**Total**	**Mild**	**Moderate**	**Severe**	**Total**	**Mild**	**Moderate**	**Severe**
No	1	1	1	1	1	1	1	1
Yes	4.250 (2.369–7.626)	1.686 (0.978–2.908)	2.653 (1.526–4.611)	2.006 (1.221–3.297)	4.029 (2.217–7.322)	1.537 (0.880–2.686)	2.563 (1.443–4.552)	1.84 (1.100–3.077)
	*p* < 0.001*	*p* = 0.06	*p* = 0.001*	*p* = 0.006*	*p* < 0.001*	*p* = 0.131	*p* = 0.001*	*p* = 0.02*

Results were from logistic regression analysis of the relations between anxiety and incidence of exacerbation in one months. Results were expressed as odds ratio (95% confidence interval). Multivariable adjustment included age, gender, BMI, COPD course, smoking history and exacerbations in the previous year.

*Results were considered significant when *p* < 0.05.

**TABLE 6:** Anxiety in relation to frequency of different levels of acute exacerbations in COPD.

	Acute exacerbation in 12 months							
**Anxiety**			**Unadjusted IRR (95% CI)**				**Multivariable adjusted IRR (95% CI)**	
	**Total**	**Mild**	**Moderate**	**Severe**	**Total**	**Mild**	**Moderate**	**Severe**
No	1.00	1.00	1.00	1.00	1.00	1.00	1.00	1.00
Yes	2.000 (1.572–2.545)	1.495 (0.922–2.424)	2.285 (1.422–3.669)	2.080 (1.350–3.203)	1.954 (1.537–2.485)	1.353 (0.839–2.181)	1.969 (1.200–3.233)	1.915 (1.227–2.989)
	*p* < 0.001*	*p* = 1.492	*p* = 0.001*	*p* = 0.001*	*p* < 0.001*	*p* = 0.215	*p* = 0.007*	*p* = 0.004*

Multivariable Poisson regression model was used for the frequency of future acute exacerbation in one year. Results were expressed as incidence-rate ratios (95% confidence interval). Multivariable adjustment included age, gender, BMI, COPD course, smoking history and exacerbations in the previous year.

*Results were considered significant when *p* < 0.05.

Multivariable adjusted odds ratio (95% CI) of future acute exacerbation in older COPD patients with anxiety was 4.029 (2.217–7.322) compared to those without anxiety. Multivariable adjusted odds ratios (95% CI) were 2.563 (1.443–4.552) and 1.84 (1.100–3.077) for moderate and severe acute exacerbations in one year (Table 5). Meanwhile, Multivariable adjusted incidence-rate ratio (95% CI) for total acute exacerbation was 1.954 (1.537–2.485) in older COPD patients with anxiety compared to those without anxiety. Corresponding incidence-rate ratios (95% CI) were 1.969 (1.200–3.233) and 1.915 (1.227–2.989) for moderate and severe acute exacerbations within one year after additional adjustment for potential confounders (Table 6).

## 4 Discussion

Chronic obstructive pulmonary disease (COPD) usually coexists with various comorbidities. Anxiety, an important comorbidity of COPD, is frequently under-diagnosed and significantly impacts the prognosis of COPD patients, especially in older COPD patients.

In our analysis, the prevalence of anxiety in older COPD patients was 19.81%. However, different studies had reported various prevalence rates. For instance, a cross-sectional study conducted in Shanghai included 275 mild COPD patients from urban communities and found that 7.6% had anxiety (21). Another study evaluated 491 Chinese COPD patients by Hospital Anxiety and Depression Scale (HADS) and reported an anxiety prevalence rate of 10% (22). The China Pulmonary Health Study (CPH) revealed that anxiety affected approximately 10.79% COPD patients (23). This discrepancy could be attributed to differences sample size, methodological design, participant sources, screening instruments, and severity levels of COPD (24).

Chronic obstructive pulmonary disease (COPD) primarily affected older populations and exhibited male predominance; this trend was also evident in our cohort where there were more male participants. However, no gender differences were observed between the two groups. Our findings indicated that COPD patients with anxiety tended to have higher pack-years, have greater comorbidities, and experience more frequent exacerbations. Additionally, COPD patients with anxiety exhibited higher levels of dyspnea (mMRC), worse health status (CAT score), and less exercise capacity (6MWD). Our analysis indicated that the BODE index, mMRC score and CAT score were associated with anxiety (*P* < 0.05).

It is worth noting that anxiety negatively impacts COPD. On one hand, the symptoms of COPD, such as gradually increasing dyspnea, cough, and expectoration, may be the main cause of anxiety in COPD patients (25). On the other hand, other comorbidities such as lung cancer, cardiovascular disease, and gastroesophageal reflux disease contribute to the occurrence of anxiety in COPD patients. Our study revealed that an increased comorbidities was associated with a higher risk of anxiety (OR 5.671; 95% CI: 3.193–10.07). Acute exacerbation of COPD is associated with increased mortality rate (26, 27). 25% of patients experiencing acute exacerbation was required for ICU admission, further increasing the economic burden of COPD (28). Additionally, frequent acute exacerbation severely worsened patients’ quality of life. A previous study conducted by our group identified that anxiety, angina, and hypertension were independent risk factors for acute exacerbation within a year (29, 30). In our study, we found that acute exacerbation in the previous year were related to anxiety and increased the risk of anxiety (OR 4.004; 95% CI: 2.204–7.273). We also discovered that older COPD patients with anxiety increased the risk of future exacerbation in one year, especially moderate and severe acute exacerbation compared to those without anxiety.

Older COPD patients with anxiety have worse dyspnea symptoms, more comorbidities, and experience more frequent acute exacerbations. Therefore, early diagnosis of COPD with anxiety is very important. However, the current scales for the diagnosis and assessment of anxiety are professional and complex. Fortunately, respiratory physicians are sensitive to clinical indicators. If there was a possibility that respiratory physicians can evaluate COPD patients with anxiety through clinical indicators, they would transfer them to psychologists as soon as possible for further treatment including psychotherapy, medications, and exercise. That would improve treatment compliance, improve symptoms, and reduce acute exacerbations of older COPD with anxiety.

There are some limitations to consider regarding our study findings. Firstly, the data on acute exacerbations were obtained from the medical records of COPD patients. Considering that some patients may have sought treatment from other hospitals during acute exacerbation episodes, there was a possibility of underreporting the frequency of acute exacerbations. Additionally, it should be noted that different assessment tools for evaluating anxiety may yield different results. In our study, HAMA was used to assess anxiety in older COPD patients.

## 5 Conclusion

In summary, our study found that older COPD patients with anxiety had worse symptoms, more comorbidities and more frequent. In addition, our study also found anxiety can increase the risk of future acute exacerbation in older COPD patients. In COPD management, routine screening for psychiatric symptoms should be an integral part of clinical work to reduce the risk of anxiety in older COPD patients at an early stage.

## Data availability statement

The original contributions presented in this study are included in the article/supplementary material, further inquiries can be directed to the corresponding author.

## Ethics statement

The study was conducted in accordance with the Declaration of Helsinki, and approved by Ethic Committee of Huadong Hospital (protocol code 20180064). The studies were conducted in accordance with the local legislation and institutional requirements. The participants provided their written informed consent to participate in this study. Written informed consent was obtained from the individual(s) for the publication of any potentially identifiable images or data included in this article.

## Author contributions

YM: Methodology, Project administration, Writing – original draft, Writing – review and editing. LS: Methodology, Investigation, Writing – review and editing. YL: Software, Validation, Writing – original draft. YW: Data curation, Resources, Writing – review and editing. ZH: Methodology, Software, Writing – review and editing. XL: Validation, Writing – review and editing. HZ: Visualization, Writing – review and editing. HG: Project administration, Supervision, Writing – review and editing.
